# Parenchymal Guidewire Perforation during ERCP: An Unappreciated Injury

**DOI:** 10.1155/2015/670323

**Published:** 2015-11-26

**Authors:** M. Ezzedien Rabie, Saad Al Faris, Ali Nasser, Abdul Aziz Shahir, Yasser Al Mahdi, Mansour Youssef Al Asmari

**Affiliations:** ^1^Department of Surgery, Armed Forces Hospital, Southern Region, P.O. Box 101, Khamis Mushait, Aseer, Saudi Arabia; ^2^Department of surgery, Aseer Central Hospital, Abha, Aseer, Saudi Arabia

## Abstract

ERCP is attended with certain complications, the majority of which are well known to the medical community. Other less-known complications also exist. Guidewire injury to the hepatic or pancreatic parenchyma represents one of the much less appreciated, albeit preventable, complications. In this report, we present the clinical course of three patients who sustained guidewire perforation of the pancreatic or hepatic parenchyma. In one patient, the clinical deterioration was confidently attributed to guidewire perforation of the pancreatic parenchyma. Conservative treatment was successful and unnecessary emergency surgery was thus avoided. In the other two, in whom the cause of the clinical deterioration was unclear, an emergency surgery was performed. Guidewire injury to the hepatic parenchyma was then confirmed which needed only intraperitoneal drainage, with successful outcome.

## 1. Introduction

Endoscopic retrograde cholangiopancreatography (ERCP) is an indispensable method for relieving biliary obstruction. Yet the procedure is attended with certain complications, which may be life-threatening.

Known complications are pancreatitis, bleeding, perforation, cholangitis, cholecystitis, and cardiopulmonary complications in the form of arrhythmias, hypoxia, and aspiration [1]. In addition, rarer complications also occur [2, 3].

An unappreciated complication is hepatic or pancreatic parenchymal perforation by the guidewire, leading to severe clinical deterioration, akin to the more appreciated and serious forms of periduodenal perforations.

## 2. Case Presentation

### 2.1. First Case

A 42-year-old female presented with progressively increasing right upper quadrant pain over the past month.

On examination, she was jaundiced but otherwise looked fairly well. Her blood pressure was 112/64 mm Hg, pulse 72/min, temperature 36.8°C, and oxygen saturation 98%. Her cardiac and chest examinations were normal and her abdomen was soft with mild right hypochondrial tenderness.

Her total bilirubin was 75 *μ*mol/L (reference range 3–17), direct bilirubin 54 *μ*mol/L (reference range 0–8), alkaline phosphatase 110 U/L (reference range 36–126), alanine aminotransferase 354 U/L (reference range 10–61), aspartate aminotransferase 205 U/L (reference range 10–42), and gamma glutamyl transferase 279 U/L (reference range 5–64) with normal blood picture and renal values.

The patient was admitted as a case of obstructive jaundice for further investigations and ultrasound abdomen showed gallstones with dilatation of the common bile duct (CBD, 8 mm) and central biliary radicles.

ERCP confirmed the presence of a small distal CBD stone, which was retrieved with CBD clearance. The procedure was reported as a straightforward one.

The following day, the patient developed left hypochondrial pain, and her serum amylase became 500 U/L (reference range 27–130), but she remained well.

One day later, she became anxious and flushed. Her respiration rate became 22/min, pulse 116/min, and blood pressure 116/70 mm Hg and abdominal examination revealed moderate tenderness in the upper abdomen.

**Figure 1:**
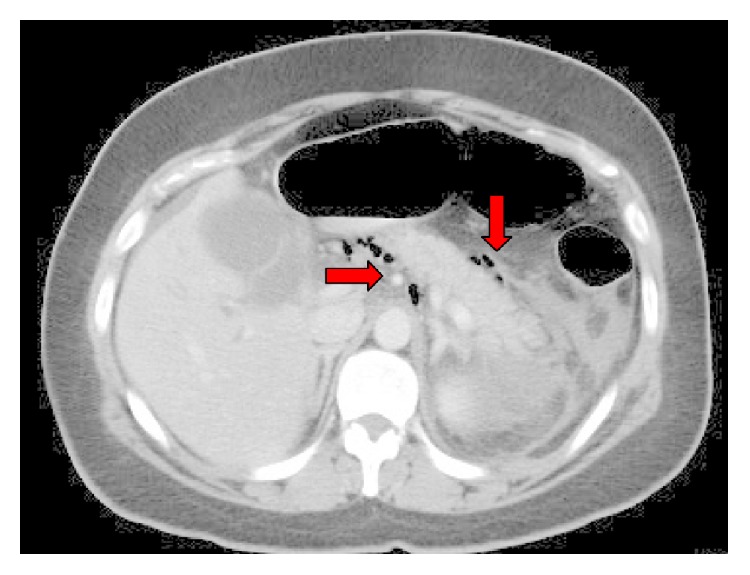
Air along the upper and lower borders of the pancreas (red arrows).

**Figure 2:**
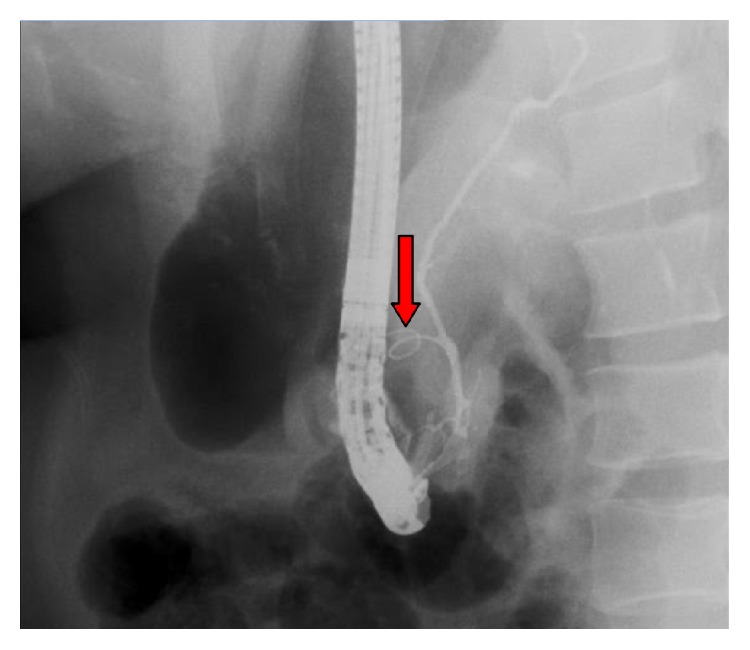
Penetration of the pancreatic duct and parenchyma by the guidewire (red arrow).

Computerized axial tomography (CT) scan showed evidence of acute pancreatitis with retroperitoneal air along the upper and lower borders of the pancreas (Figure 1) with dilated CBD and biliary radicles. Although there was no extravasation of the contrast outside the duodenum, the presence of retroperitoneal air and the clinical deterioration of the patient raised the possibility of periduodenal perforation demanding emergency surgery and preparations for exploratory laparotomy were initiated. Another careful review of the CT scan and ERCP shots by a senior radiologist recognized the presence of pancreatic parenchymal perforation by the guidewire (Figure 2), a situation which does not demand surgery. Consequently, a conservative approach was initiated.

The patient was admitted to the ICU, where she was kept on nil orally with IV fluids and antibiotics. In the ensuing days, although her blood pressure remained stable, she developed high fever (41°C) and tachycardia (140/min) with low O_2_ saturation (90% on nonrebreathing face musk 15 L/min). Chest X-ray showed bilateral lung infiltrate and pleural effusion, suggestive of ARDS; for that reason she needed intubation and ventilation with inotropic support.

For follow up, CT showed pancreatic tail necrosis with free fluid in the abdomen and decrease in the amount of retroperitoneal air.

After a turbulent course in the ICU, the patient improved and was extubated and finally discharged home. Two months later, when a control CT scan showed no abnormality, unremarkable laparoscopic cholecystectomy was performed and the patient was discharged in good condition and remained so on follow up.

### 2.2. Second Case

A 28-year-old female, with past history of hypothyroidism on treatment and splenectomy for unknown reason, presented with a three-day history of upper abdominal pain of sudden onset, vomiting, and dark urine.

Her examination was unremarkable apart from right hypochondrium tenderness and guarding.

Her blood works showed raised white cell count of 16.200/mm^3^ (reference range 4.000–11.000) and normal amylase and renal values, while her liver functions were deranged. Alkaline phosphatase was 353 U/L (reference range 36–126), alanine aminotransferase 381 U/L (reference range 10–61), aspartate aminotransferase 230 U/L (reference range 10–42), gamma glutamyl transferase 467 U/L (reference range 5–64), and total bilirubin 71 *μ*mol/L (reference range 3–17).

Abdominal ultrasound showed multiple gallstones with thick walled gallbladder; for that reason ERCP was performed to retrieve a small CBD stone. The procedure was reported to be unremarkable.

Following ERCP, abdominal pain and tenderness increased alarmingly; for that reason CT scan was performed to show free fluid around the liver and in the right hepatorenal pouch. Iatrogenic ERCP related injury was suspected and the patient was prepared for laparoscopic exploration/exploratory laparotomy.

**Figure 3:**
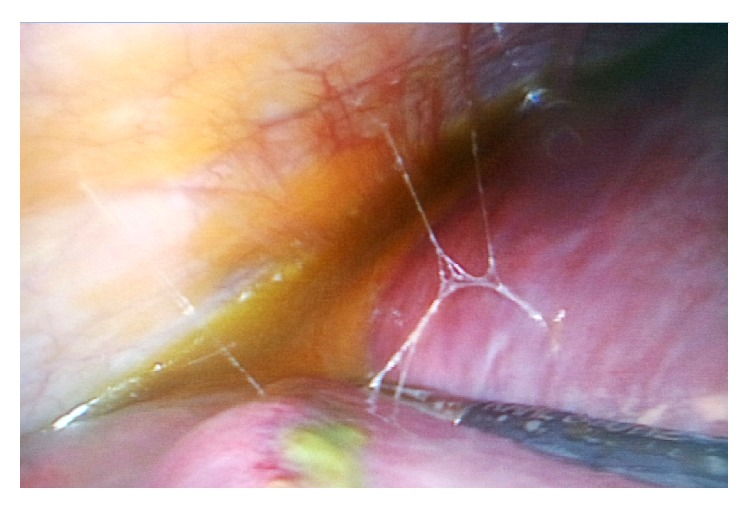
Bile collection between the upper surface of the right hepatic lobe and diaphragm.

**Figure 4:**
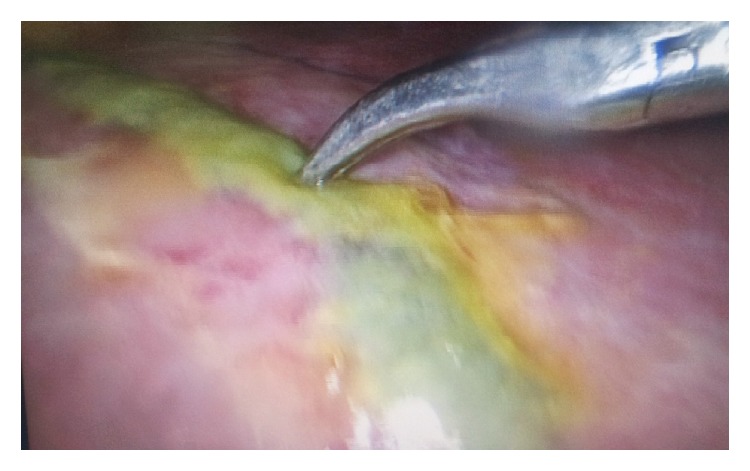
Bile stained tissue on the superior surface of the liver.

On laparoscopy, free bile was found around the liver, with areas of necrotic looking and bile stained tissue on the anterosuperior surface of the liver (Figures 3 and 4). The gallbladder was found acutely inflamed but intact with no evidence of perforation. Bile was aspirated and straightforward cholecystectomy was then performed. During the procedure, tissues in Calot's triangle as well as the neighbouring tissues appeared clean. Guidewire perforation of the anterosuperior liver surface was then assumed as the source of bile leak. An intraperitoneal drain was inserted and the procedure was concluded.

Postoperatively, antibiotics continued and the patient tolerated surgery well with uneventful recovery, when she was discharged for outpatient follow up.

On her follow up visit, she remained well and histopathology showed acutely inflamed gallbladder.

### 2.3. Third Case

A 75-year-old male presented with obstructive jaundice two weeks earlier to his latest presentation. At that time, ERCP was done and a stone was retrieved from the CBD. Cholecystectomy was offered to the patient but he refused, asking for more time for pondering. Two weeks later, he presented again with upper abdominal pain, tenderness, fever, and deranged liver enzymes, suggestive of biliary obstruction. Another ERCP was performed and a CBD stone was again extracted.

Following ERCP, abdominal pain intensified with fever (>38°C) and leucocytosis (16.000/mm^3^, reference range 4.0–11.0) developing, findings suggestive of ERCP related injury. Abdominal CT, which showed little amount of air around the right kidney, with free fluid in the left upper quadrant, confirmed this suspicion.

After the necessary preparations, an emergency laparotomy was performed. On entering the abdomen, free bile was found in the left upper quadrant and an indurated part of greater omentum near the splenic flexure was found encasing a collection of bile. Culture swabs were taken and free bile was aspirated.

**Figure 5:**
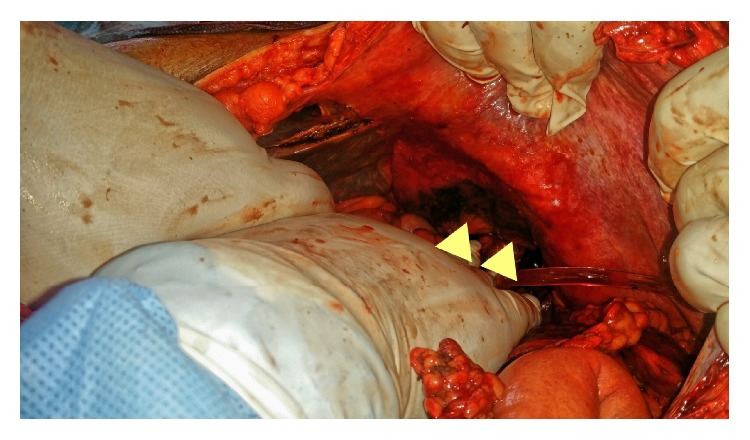
A longitudinal band of bile stained tissue on the under surface of the left cupola of the diaphragm.

The liver surfaces of both hepatic lobes were found fixed to the under surface of the diaphragm with dense and apparently old adhesions, and there was a localized bile stained area on the under surface of the left hemidiaphragm (Figure 5).

**Figure 6:**
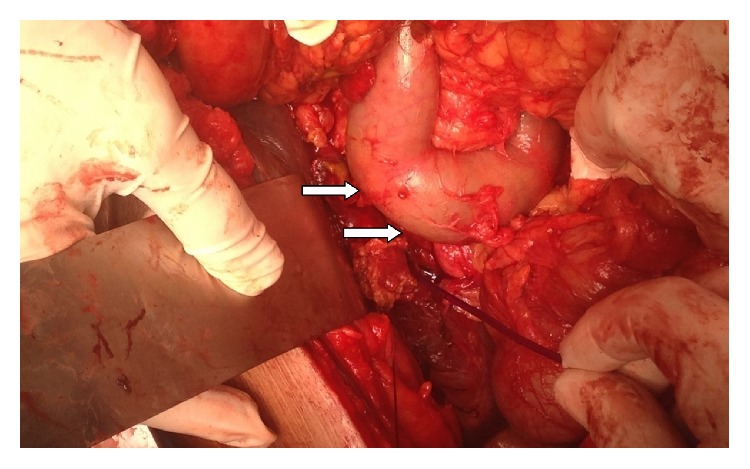
A fully kocherised duodenum (white arrows) with no evidence of injury and no bile stain in the surrounding retroperitoneum.

A meticulous search was then conducted for the source of bile leak, which entailed mobilization of both colonic flexures with full kocherisation of the duodenum. This failed to show the source as there was no duodenal perforation (Figure 6) or soiling of the retroperitoneum in the right perinephric area. Additionally, methylene blue was instilled in the stomach and duodenum to check for possible minor leak and none was found.

At the conclusion, unremarkable cholecystectomy was performed and, as a last measure, methylene blue was injected through the cystic duct into the CBD to check for leak and again none was found.

Haemostasis was effected though with some difficulty due to splenic capsular injury, and thorough saline wash was done and the abdomen was closed around drains.

Postoperatively, the patient was kept in the ICU where antibiotic therapy, blood transfusion, and inotropic support were introduced. Eventually, he recovered and was discharged for outpatient follow up, where he appeared in good condition.

## 3. Discussion

The most feared complication or ERCP is periduodenal injury, for which Stapfer et al. [4] and Howard et al. [5] independently introduced similar grading systems. The grades of this injury are, in ascending order of severity, presence of retroperitoneal air, guidewire injury to the CBD, periampullary injury, and, lastly, duodenal injury away from the papilla. Other less known classification systems based on the injuring device also exist [6]. Categorizing this injury facilitates the choice of the management plan, which is usually a difficult task.

Wu et al. [7] reported on their experience with ERCP associated injuries. In their series, the rate of injury was 0.45% and most cases responded to nonoperative measures. Their mortality rate was 16.7, which occurred mainly in those who sustained duodenal and periampullary perforations. Delay in performing surgery when needed, due to either delayed diagnosis or performing surgery after a period of unsuccessful medical treatment, was associated with increased mortality [7].

In our unit, we had a periduodenal perforation rate of 1.7% (10/597 case), which carried a mortality rate of 10% (1/10 cases). Depending on the critical appraisal of the clinical and radiological findings, our management policy was individualized for each patient and most patients were treated conservatively. In our report, we suggested dividing the radiological findings into hard and soft signs. Contrast extravasation, free air in the peritoneal cavity, and periduodenal fluid collection were considered hard signs, while duodenal wall thickening, intramural duodenal air, retroperitoneal fat stranding, and free air in the retroperitoneum, mediastinum, pleural sac, or subcutaneous tissue were considered soft signs. The presence of any of the hard signs is an indication for surgery, whereas soft signs in the absence of florid clinical findings are indications for a conservative treatment [8].

In the patients presented here, liver and pancreatic parenchymal perforation by the guidewire were behind the patients' deterioration following ERCP. In the first patient, although the clinical deterioration was very much suggestive for the need for surgery, careful review of the ERCP films and CT scan enabled a confident diagnosis of pancreatic parenchymal injury to be made, and this allowed a conservative treatment to be followed, which proved successful at the end. In the other two patients with liver parenchymal injury, the situation was more complicated. In one of them, the second patient, the clinical deterioration in the presence of free fluid in the abdomen was suggestive of periduodenal perforation, demanding surgery. The presence of bile collection and stained tissue on the anterosuperior surface of the liver, in the absence of another possible source, left the liver as the sole source of leak. In the third patient, the presence of fluid in the left upper abdomen with clinical deterioration was again suggestive of periduodenal perforation, mandating surgery. At laparotomy, we were convinced that the source of bile was the liver surface, after its perforation by the guidewire. From there, bile collected between the upper surface of the liver and diaphragm, to trickle to the left upper abdomen, aided by the old dense adhesions between the liver and under surface of the diaphragm. A confirmation of this explanation is the absence of any soiling of the periduodenal or right perinephric tissues.

In this regard, it is noteworthy to mention that the location of perforation in cases of ERCP associated injury may not be identified at laparotomy, as was seen in 7 out of 30 patients in Wu series [7]. In this situation, liver parenchymal perforation by the guidewire may account for some case, and as the liver has extensive surfaces, mostly hidden from direct view, the source of leak may not be identified.

**Figure 7:**
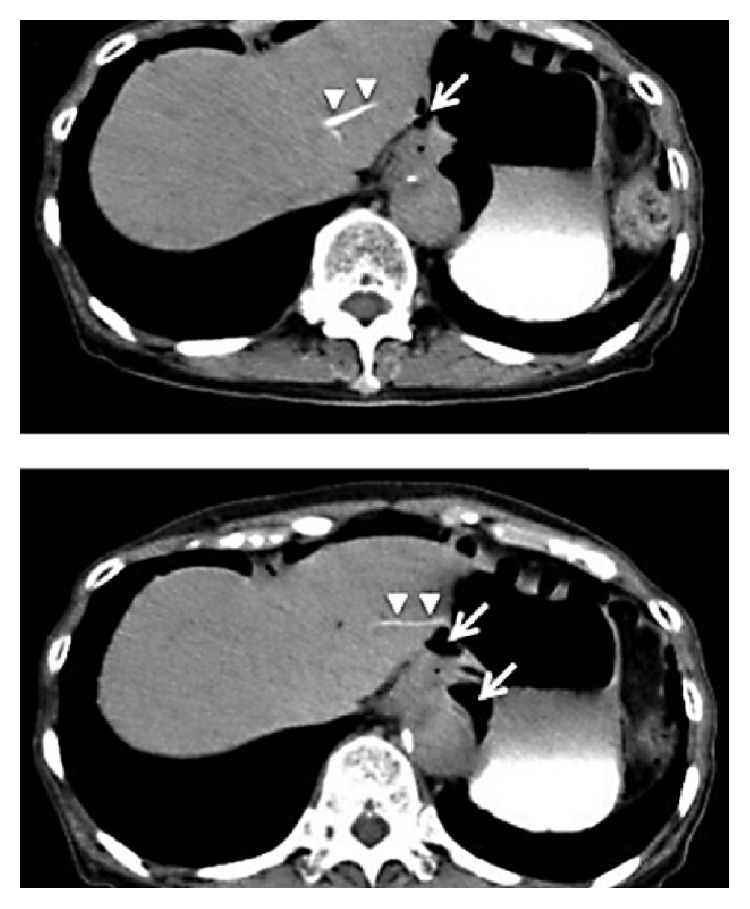
CT showing intraperitoneal air (arrow) with contrast leak from the left hepatic lobe parenchyma (arrow heads) (Kayashima et al. [9], with permission).

**Figure 8:**
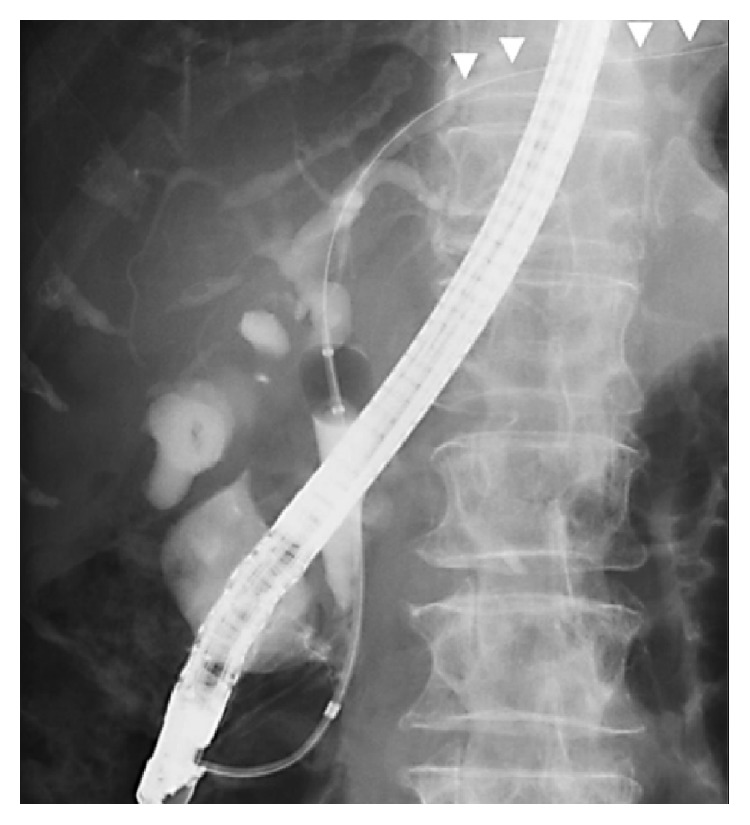
The guidewire penetrating deep into the parenchyma of the left hepatic lobe (arrow heads) (Kayashima et al. [9], with permission).

By this report, we aim at directing the attention of the surgical community as well as the gastroenterologists and radiologists to the existence of this injury, which could be easily prevented by gentle manipulation of the guidewire. The practical impact of this knowledge may appear little, as it is always safer to operate in the presence of free fluid in the abdomen following ERCP, in the relevant clinical context. However, with pancreatic parenchymal injury, once the diagnosis is made with confidence, a conservative treatment may be followed with success, as happened in the first patient. More importantly, absence of a clear source of bile leak after reasonable exploratory endeavours should lead one to assume the diagnosis of liver parenchymal injury by the guidewire and provide only peritoneal drainage, as in our second and third patients, who responded to peritoneal drainage only. Unfamiliarity with this injury leads to the provision of unneeded biliary and gastrointestinal diversions, with their attended complications and prolongation of hospital stay. In a report by Kayashima et al. [9], despite the solid proof they had (Figures 7 and 8), needless T tube drainage of the CBD with tube duodenostomy was performed.

However, as previously mentioned, delaying needful surgery is associated with increased mortality [7], one should exercise caution when considering the diagnosis of parenchymal guidewire perforation. In this regard and although difficult to conceive, ERCP performed following such injury may demonstrate contrast leak from the peripheral bile ducts at the site of injury [10].

Our Medline search, using the terms liver, parenchyma, guidewire, and perforation in the title and abstract, in different combinations, yielded no relevant results. This might reflect either the rarity of the condition or, more probably, the unawareness of its existence. In this context, rare related injuries have been also reported. Subcapsular liver haematoma may follow ERCP [10–13], with 16 cases reported in the literature in the year 2011 [11], and subcapsular biloma was also very rarely reported [14].

In Table 1, we propose a simple classification for this injury and its possible consequences.

## 4. Conclusion

Liver and pancreatic parenchymal perforation by the guidewire during ERCP is an unappreciated complication which leads to severe clinical deterioration, akin to the more serious forms of ERCP associated periduodenal perforations. It should be avoided by gentle handling of the guidewire during the procedure. Careful review of the ERCP shots and CT scan, done immediately after ERCP, may enable the diagnosis to be made. When diagnosed with confidence, it allows for conservative treatment to be followed, while the gall bladder could be removed later. If discovered upon surgery, it needs only peritoneal drainage with no biliary or gastrointestinal diversions, in addition to cholecystectomy.

## Figures and Tables

**Table 1:** Subtypes of parenchymal guidewire injury with possible consequences.

Subtype	Location
A	Pancreatic duct into parenchyma^*∗*^
B	Pancreatic duct through parenchyma^*∗*^
C	Intrahepatic bile ducts into parenchyma^*∗∗*^
D	Intrahepatic bile ducts through parenchyma^*∗∗∗*^

^*∗*^Both types A and B may result in peripancreatic fluid collection due to pancreatitis alone (type A) or pancreatitis and leak from the pancreatic duct (type B).

^*∗∗*^Subcapsular haematoma or biloma results.

^*∗∗∗*^Free bile leak results.
